# Malassezia (Pityrosporum) Folliculitis Incognito: Malessezia-associated Folliculitis Masked by Topical Corticosteroid Therapy

**DOI:** 10.7759/cureus.6531

**Published:** 2020-01-01

**Authors:** Philip R Cohen, Christof Erickson, Antoanella Calame

**Affiliations:** 1 Dermatology, San Diego Family Dermatology, San Diego, USA; 2 Dermatology, Compass Dermatopathology, Inc., San Diego, USA; 3 Dermatology/Dermatopathology, Compass Dermatopathology, Inc., San Diego, USA

**Keywords:** corticosteroid, folliculitis, incognito, malassezia, papule, pityrosporum, pustule, scabies, tinea, topical

## Abstract

Malassezia (Pityrosporum) folliculitis usually appears as pruritic monomorphous papules and pustules on the upper back, chest, extensor arms and face. Acne vulgaris, bacterial folliculitis, eosinophilic folliculitis and systemic corticosteroid-induced acne can clinically mimic the fungal-caused acneiform condition. The designation incognito is used to describe tinea or scabies when the characteristic presentation is masqueraded by the application of topical corticosteroid treatment. Application of corticosteroid cream altered the morphology of the skin lesions in a man with Malassezia folliculitis. His cutaneous findings-localized areas of post-inflammatory hyperpigmentation with flattened or completely resolved follicular papules-raised the possibility of partially treated follicular eczema or follicular contact dermatitis. Pathognomonic findings from biopsies of the skin lesions established the diagnosis of Malassezia folliculitis; the condition completely resolved after treatment with topical antifungal shampoo and cream. Similar to tinea incognito and scabies incognito, folliculitis caused by Malassezia yeast in which the cutaneous morphology has been concealed by management with topical corticosteroids should be referred to as Malassezia (Pityrosporum) folliculitis incognito.

## Introduction

Malassezia folliculitis is an acneiform condition of fungal etiology. It has previously been referred to as Pityrosporum folliculitis. Malassezia folliculitis typically presents as pruritic follicular papules and pustules on the upper torso and face [1].

Topical corticosteroids are frequently used to treat dermatologic conditions. Indeed, the application of corticosteroids is often initiated as empirical therapy when a diagnosis of dermatitis is suspected. However, the application of topical corticosteroids to cutaneous dermatophyte infection or mite infestation can alter the morphologic appearance of the dermatosis and thereby conceal the diagnosis [2,3].

A man with Malassezia folliculitis, in whom the diagnosis was masked by topical corticosteroid treatment, is described.

## Case presentation

A healthy immunocompetent 44-year-old man had a 10-year history of a recurrent, occasionally itchy, skin rash on his upper back and abdomen with no improvement on moisturizing creams or topical antibiotic ointment that had been prescribed by his previous physicians. He currently presented for a total body skin check. Examination showed individual and grouped follicular non-inflammatory papules; the clinical differential diagnosis included follicular eczema and follicular contact dermatitis for the back and abdomen lesions, respectively. Triamcinolone acetonide 0.1% cream, twice daily for seven days, resulted in significant improvement of the dermatosis; however, the condition would promptly recur after treatment.

**Figure 1:**
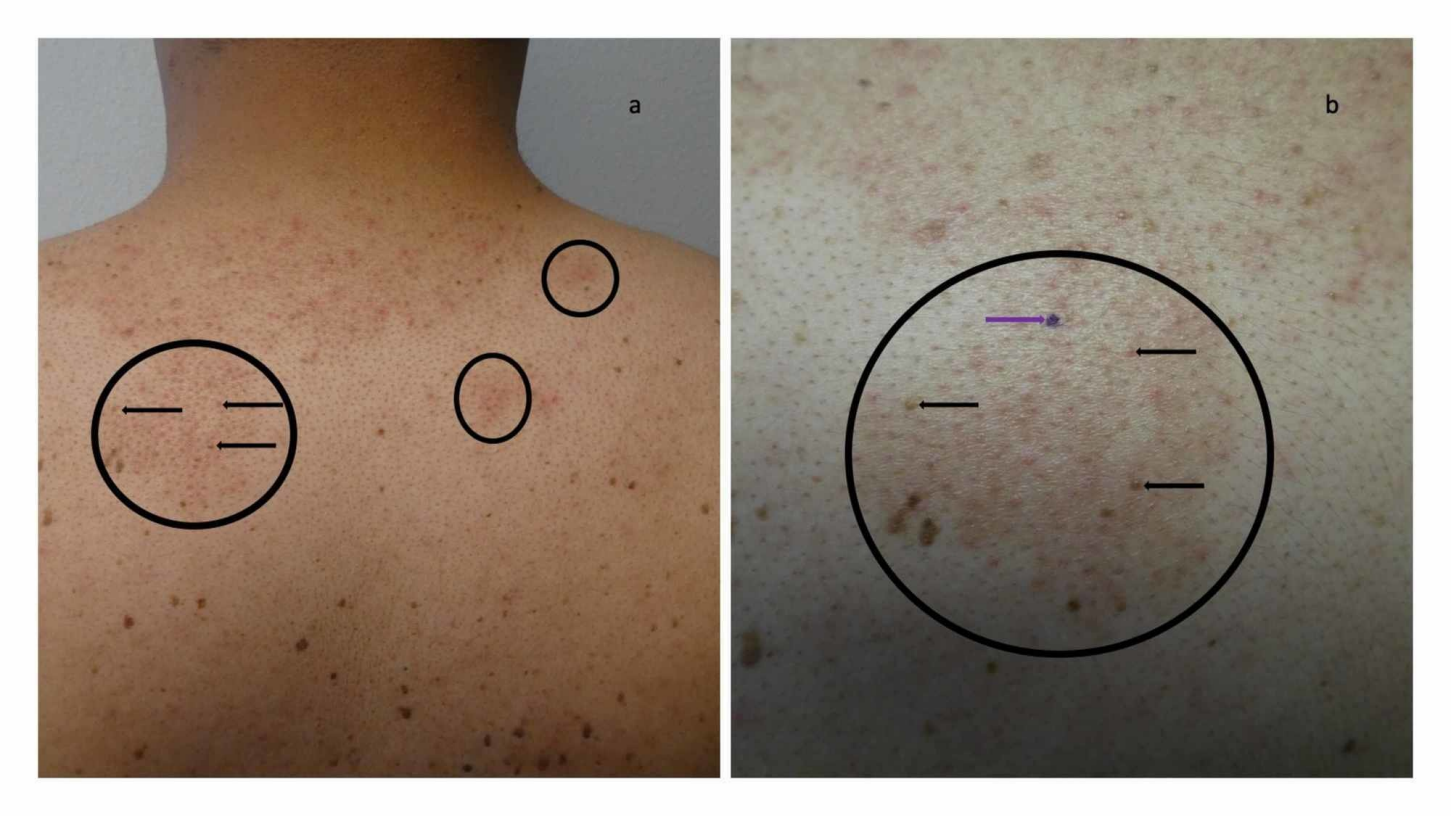
Malassezia (Pityrosporum) folliculitis incognito on the posterior neck and upper back of a 44-year-old man mimicking treated follicular eczema Distant (a) and closer (b) views of the posterior neck and upper back show several areas (black circles) in which follicular papules have flattened or completely resolved (horizontal black arrows pointed towards the left). In addition to prominent hair follicles, there is diffuse brown pigmentation of the skin consistent with post-inflammatory hyperpigmentation. The purple mark (b) is the biopsy site (purple arrow pointing towards the right).

**Figure 2:**
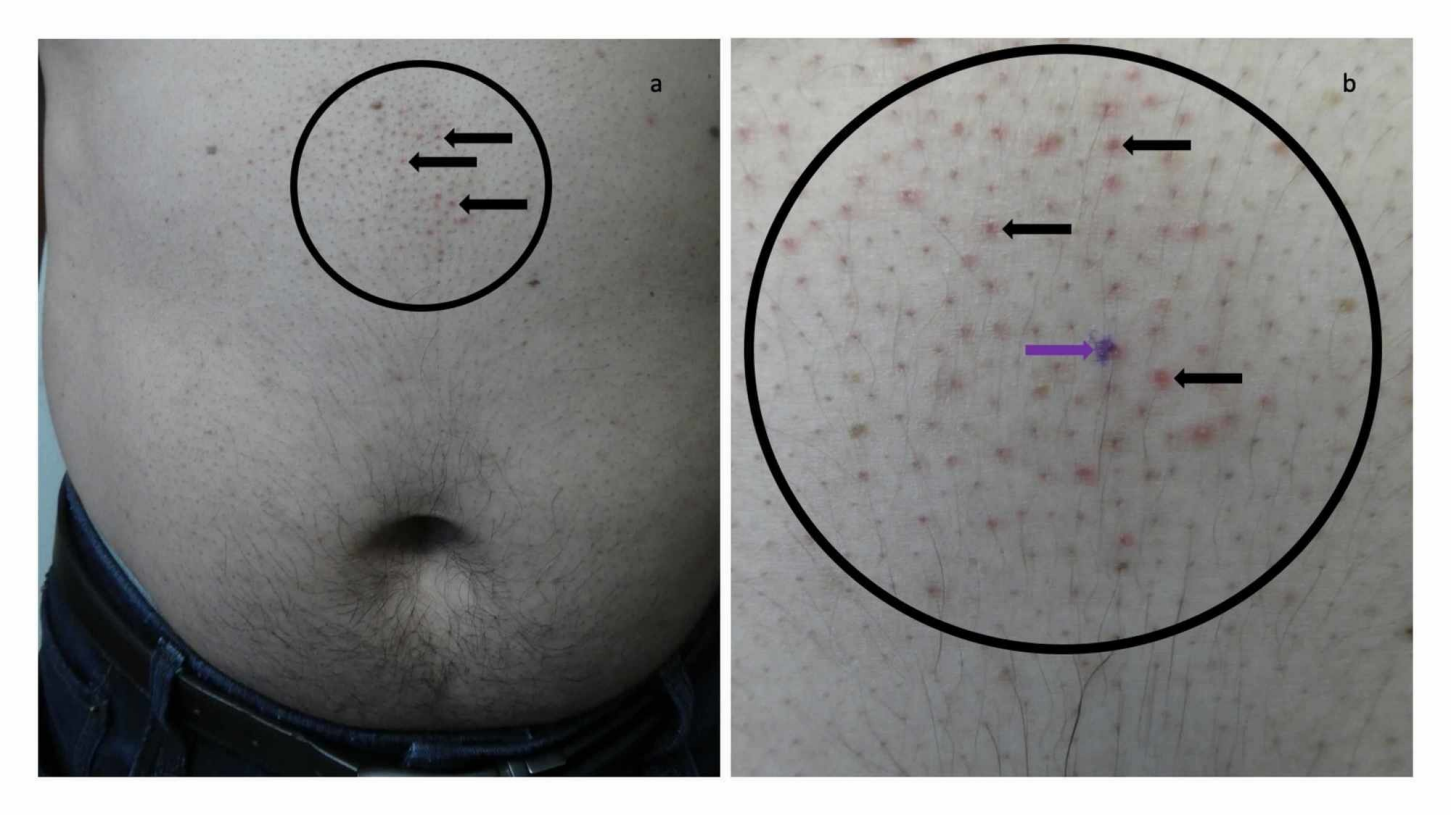
Malassezia folliculitis incognito on the abdomen of a 44-year-old man mimicking treated follicular contact dermatitis Distant (a) and closer (b) views of the mid abdomen above the umbilicus shows an area (black circle) in which follicular papules have flattened or completely resolved (horizontal black arrows pointed towards the left) and prominent hair follicles. The purple mark (b) is the biopsy site (purple arrow pointing towards the right).

The patient subsequently presented for evaluation of his skin condition after recently completing a week of topical therapy with the corticosteroid cream. Cutaneous examination demonstrated localized areas of flattening or completely resolved follicular papules not only on the posterior neck and upper back (Figure 1), but also on the mid abdomen above his umbilicus (Figure 2); the individual hair follicles in the areas were also prominent. In addition, there was also brown darkening of the surrounding skin at prior sites of the condition on his back, consistent with post-inflammatory hyperpigmentation.

Similar to his initial presentation, the differential diagnosis included treated follicular eczema (on the upper back) and follicular contact dermatitis (on the abdomen). However, since the prior treatment with topical corticosteroids may have partially resolved the inflammatory component of the patient’s condition, the possibility of a primary folliculitis (either associated with bacteria, eosinophils, or yeast) was also considered. Therefore, punch biopsies from the upper back and the abdomen were performed. Both biopsies showed similar pathologic changes. 

**Figure 3:**
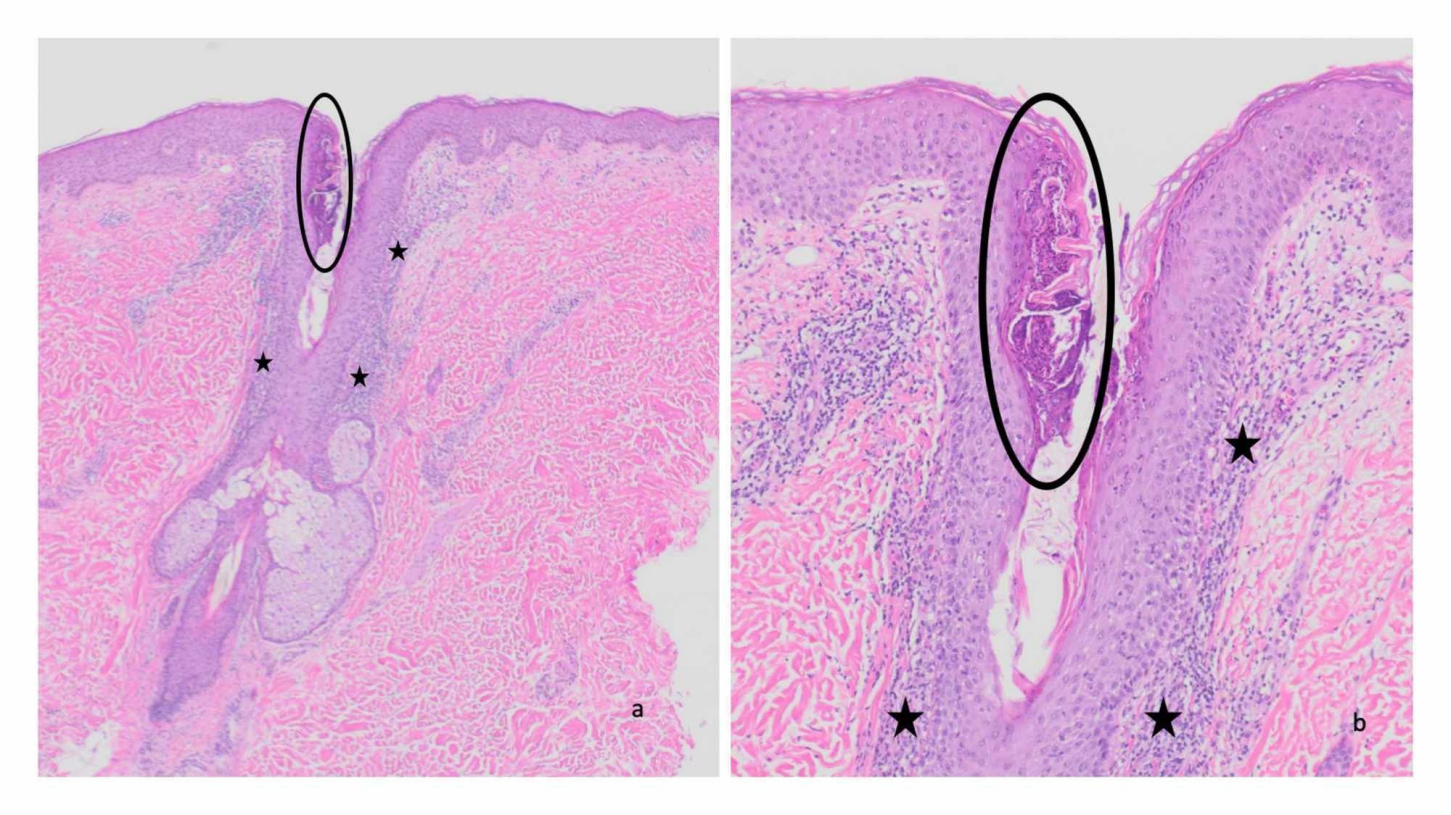
Pathologic changes of Malassezia folliculitis—hematoxylin and eosin stained sections Distant (a) and closer (b) views of the biopsy specimen from the abdomen show inflammation in the dermis (black stars) consisting of neutrophils and lymphocytes adjacent to a hair follicle. A collection of neutrophils and yeast are present in the upper portion of the hair follicle (black circle). Hematoxylin and eosin: a, x40; b, x100.

**Figure 4:**
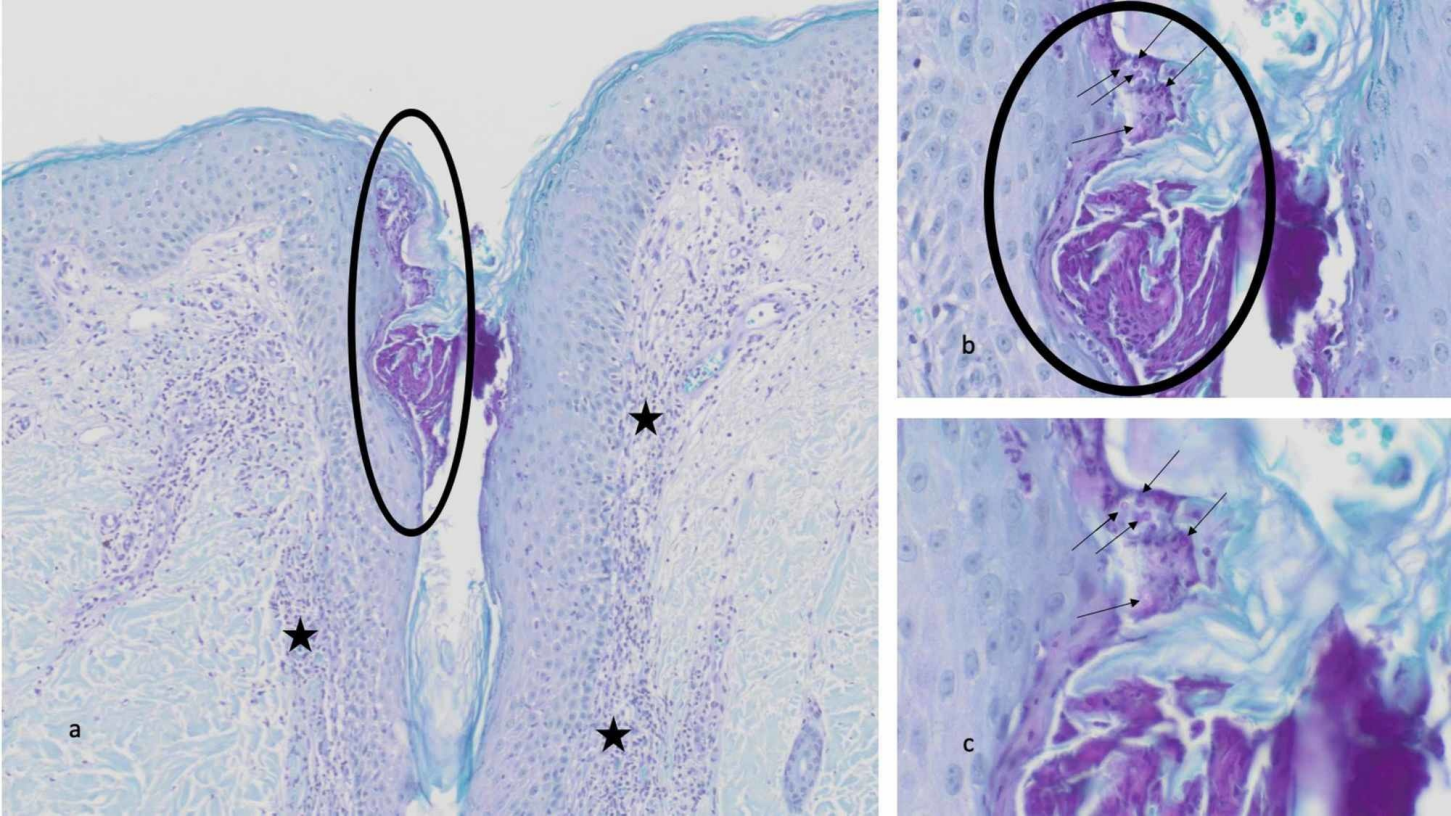
Pathologic changes of Malassezia folliculitis—periodic acid-Schiff stained sections Distant (a) and closer (b and c) views of the biopsy specimen from the abdomen show a perifollicular infiltrate of neutrophils and lymphocytes in the dermis (black stars). The infundibulum of the hair follicle contains a collection of neutrophils and yeast (black circle). Individual fungal organisms appear as round purple spores (black arrows). Periodic acid-Schiff: a, x100; b, x400; c, x600.

Microscopic examination of the biopsy specimen taken from the abdomen and stained with hematoxylin and eosin showed a prominent hair follicle with a perifollicular infiltrate of neutrophils and lymphocytes in the dermis (Figure 3). Within the upper portion of the hair follicle, the infundibulum, there is a collection of neutrophils and numerous yeast. A periodic acid-Schiff stain highlights the fungal elements within the follicle as round purple organisms (Figure 4).

The pathologic changes observed are those of Malassezia folliculitis. The topical application of the corticosteroid cream accounted for the atypical morphologic appearance of the skin condition. Therefore, correlation of the history, clinical presentation and pathology findings established the diagnosis of Malassezia (Pityrosporum) folliculitis incognito.

The patient was treated for four weeks with ketoconazole 2% shampoo to the affected areas in the shower for five minutes each day and ketoconazole 2% cream twice daily to the skin lesions. There was complete resolution of the condition (with residual post-inflammatory hyperpigmentation) on follow-up. He will continue to use the topical antifungal therapies two to three times weekly to prevent recurrence.

## Discussion

Malassezia is a lipophilic yeast. Fourteen species of Malassezia have been described in humans. Malassezia furfur includes both Pityrosporum ovale and Pityrosporum orbiculare. Malassezia has been associated several skin conditions, such as atopic dermatitis, folliculitis, pityriasis (tinea) versicolor and seborrheic dermatitis [1,4,5].

An early description of Malassezia folliculitis by Weary et al. in 1969 described a 36-year-old woman with a recurrent acneiform eruption resulting from antibiotic administration [6]. Similar to the reported patient, her skin condition became pruritic and she was treated with a topical corticosteroid lotion; within 48 hours, there was substantial resolution of the eruption [6]. Four years later, in 1973, Potter et al. published a case series of seven patients with Malassezia folliculitis and established the relationship of the skin condition with the presence of Pityrosporum organisms [7].

Patients with Malassezia folliculitis may have concurrent skin conditions; these include not only other dermatoses associated with the organism, but also acne vulgaris, atopic dermatitis, seborrheic dermatitis, systemic corticosteroid-induced acne and tinea versicolor [5]. Predisposing factors for Malassezia folliculitis include excessive sweating, hot and humid climates, immunosuppression, systemic corticosteroid use and topical or oral antibiotic use; however, it can also occur in immunocompetent individuals [1,8]. Nearly 80% of the patients with the condition experience pruritus [4]. 

Malassezia folliculitis appears as monomorphous papules and pustules [1,4,5]. The most frequent lesion locations include the upper back, chest and extensor arms; lesions are also commonly found on the chin and the malar regions of the face [4]. The material obtained from squeezing a follicle-associated papule or pustule typically contains at least 10 yeasts [9]. Microscopic evaluation of a lesion skin biopsy shows round and budding yeast cells predominantly in the upper part of a dilated hair follicle and also in the central part of the follicle [10]. 

The clinical differential diagnosis of Malassezia folliculitis includes acne vulgaris, bacterial folliculitis, eosinophilic folliculitis, and systemic corticosteroid-induced acne [1,4]. The reported patient’s lesions on his initial examination were not only located on the upper back, but also on his periumbilical region; the hyperpigmentation on his back and the abdominal site of latter site were not typical features of Malassezia folliculitis. Therefore, the possibilities of follicular eczema and follicular contact dermatitis were also considered as possible diagnoses for his condition. In addition, the skin lesions promptly responded to treatment with topical corticosteroids, supporting the presumptive diagnoses [11,12].

However, recurrence of the lesions after discontinuing topical corticosteroid therapy prompted consideration of other cutaneous disorders such as folliculitis [13]; however, the morphology of the lesions after topical corticosteroid treatment was not characteristic of the typical appearance of Malassezia folliculitis. Hence, to further investigate the condition, skin biopsies of the lesions were performed. The discovery of numerous yeasts in the hair follicle with surrounding inflammation established the diagnosis of Malassezia folliculitis. In addition, the lesions completely resolved after topical antifungal shampoo (daily) and cream (twice daily); the patient will continue to use the medications at least twice weekly to prevent recurrence.

Tinea is a superficial dermatophyte skin infection. Tinea incognito refers to this cutaneous fungal infection in individuals whose lesions have atypical morphology since they have been topically treated with an immunosuppressive medication [14]. The condition was initially reported by Ives and Marks in 1968 when they described 14 patients whose clinical presentation of dermatophyte infection was bizarre and difficult to diagnose following the application of a topical corticosteroid [2].

Scabies is an infestation of the skin by the Sarcoptes scabiei mite. Orkin introduced the term scabies incognito, as an adaptation of tinea incognito, in 1975 to describe the unusual clinical presentation of the mite infestation in patients who had been treated with topical (or systemic) corticosteroids [3]. Recently, the comprehensive designation scabies surrepticius has been used for not only patients with scabies incognito, but also individuals with other atypical presentations of scabies [15].

Topical corticosteroid treatment of Malassezia folliculitis can result in a clinical morphology of the lesions that is not classic in appearance. Weary et al.’s patient’s lesions mimicked acne and the reported patient’s condition masqueraded as follicular eczema and follicular contact dermatitis [6]. Following the established convention by earlier investigators when describing patients with either tinea or scabies whose lesions have been altered following application of topical corticosteroids, it is reasonable to refer to the atypical presentation of this yeast-associated condition in patients who have been treated with topical corticosteroids as Malassezia folliculitis incognito.

## Conclusions

Malassezia folliculitis can not only be associated with acne vulgaris, atopic dermatitis, seborrheic dermatitis, systemic corticosteroid-induced acne and tinea versicolor, but also mimic several conditions including acne vulgaris, bacterial folliculitis, eosinophilic folliculitis, follicular contact dermatitis, follicular eczema, and systemic corticosteroid-induced acne. Tinea incognito and scabies incognito refer to dermatophyte infection and scabies infestations that present with skin lesions of altered morphology secondary to management with topical corticosteroids. Similar to the incognito designation that has been established when the diagnosis of either tinea or scabies is concealed by topical corticosteroid application, it is suggested to designate Malassezia-associated folliculitis that has been masked by therapy with topical corticosteroids as Malassezia (Pityrosporum) folliculitis incognito.

## References

[1] Rubenstein RM, Malerich SA (2014). Malassazia (pityrosporum) folliculitis. J Clin Aesthet Dermatol.

[2] Ive FA, Marks R (1968). Tinea incognito. Br Med J.

[3] Orkin M (1975). Today’s scabies. JAMA.

[4] Durdu M, Guran M, Ilkit M (2013). Epidemiological characteristics of Malassezia folliculitis and use of the May-Grunwald-Giemsa stain to diagnose the infection. Diagn Microbiol Infect Dis.

[5] Gaitanis G, Velegraki A, Mayser P, Bassukas ID (2013). Skin diseases associated with Malassezia yeasts: facts and controversies. Clin Dermatol.

[6] Weary PE, Russell CM, Butler HK, Hse YT (1969). Acneform eruption resulting from antibiotic administration. Arch Dermatol.

[7] Potter BS, Burgoon CF Jr, Johnson WC (1973). Pityrosporum folliculitis. Report of seven cases and review of the Pityrosporum organism relative to cutaneous disease. Arch Dermatol.

[8] Viana de Andrade AC, Pithon MM, Oiticica OM (2013). Pityrosporum folliculitis in an immunocompetent patient: clinical case description. Dermatol Online J.

[9] Suzuki C, Hase M, Shimoyama H, Sei Y (2016). Treatment outcomes for Malassezia folliculitis in the dermatology department of a university hospital in Japan. Med Mycol J.

[10] Back O, Faergemann J, Hornqvist R (1985). Pityrosporum folliculitis: a common disease of the young and middle-aged. J Am Acad Dermatol.

[11] Silverberg NB (2017). Typical and atypical clinical appearance of atopic dermatitis. Clin Dermatol.

[12] Cohen PR (2014). Follicular contact dermatitis revisited: a review emphasizing neomycin-associated follicular contact dermatitis. World J Clin Cases.

[13] Luelmo-Aguilar J, Santandreu M (2004). Folliculitis: recognition and management. Am J Clin Dermatol.

[14] Arenas R, Morena-Coutino G, Vera L, Welsh O (2010). Tinea incognito. Clin Dermatol.

[15] Cohen PR (2017). Scabies masquerading as bullous pemphigoid: scabies surrepticius. Clin Cosmet Investig Dermatol.

